# Development and Validation of Automated Visual Field Report Extraction Platform Using Computer Vision Tools

**DOI:** 10.3389/fmed.2021.625487

**Published:** 2021-04-29

**Authors:** Murtaza Saifee, Jian Wu, Yingna Liu, Ping Ma, Jutima Patlidanon, Yinxi Yu, Gui-Shuang Ying, Ying Han

**Affiliations:** ^1^Department of Ophthalmology, University of California, San Francisco, San Francisco, CA, United States; ^2^Beijing Ophthalmology and Visual Science Key Lab, Beijing Tongren Eye Center, Beijing Tongren Hospital, Beijing Institute of Ophthalmology, Capital Medical University, Beijing, China; ^3^Department of Ophthalmology, Shandong Provincial Hospital, Shandong First Medical University, Jinan, China; ^4^Department of Ophthalmology, Bhumibol Adulyadej Hospital, Bangkok, Thailand; ^5^Center for Preventive Ophthalmology and Biostatistics, Perelman School of Medicine, University of Pennsylvania, Philadelphia, PA, United States; ^6^Ophthalmology Section, Surgical Service, San Francisco Veterans Affairs Medical Center, San Francisco, CA, United States

**Keywords:** glaucoma, visual field, neuroophthalmogy, optical character reader, computer vision and image processing

## Abstract

**Purpose:** To introduce and validate hvf_extraction_script, an open-source software script for the automated extraction and structuring of metadata, value plot data, and percentile plot data from Humphrey visual field (HVF) report images.

**Methods:** Validation was performed on 90 HVF reports over three different report layouts, including a total of 1,530 metadata fields, 15,536 value plot data points, and 10,210 percentile data points, between the computer script and four human extractors, compared against DICOM reference data. Computer extraction and human extraction were compared on extraction time as well as accuracy of extraction for metadata, value plot data, and percentile plot data.

**Results:** Computer extraction required 4.9-8.9 s per report, compared to the 6.5-19 min required by human extractors, representing a more than 40-fold difference in extraction speed. Computer metadata extraction error rate varied from an aggregate 1.2-3.5%, compared to 0.2-9.2% for human metadata extraction across all layouts. Computer value data point extraction had an aggregate error rate of 0.9% for version 1, <0.01% in version 2, and 0.15% in version 3, compared to 0.8-9.2% aggregate error rate for human extraction. Computer percentile data point extraction similarly had very low error rates, with no errors occurring in version 1 and 2, and 0.06% error rate in version 3, compared to 0.06-12.2% error rate for human extraction.

**Conclusions:** This study introduces and validates hvf_extraction_script, an open-source tool for fast, accurate, automated data extraction of HVF reports to facilitate analysis of large-volume HVF datasets, and demonstrates the value of image processing tools in facilitating faster and cheaper large-volume data extraction in research settings.

## Introduction

Within ophthalmology, large volume data analysis requires structured data to perform. Data extraction and structuring are often a critical and overlooked aspect of such projects. Especially with the advent of machine learning and other “large data” processing techniques, there is a strong need for fast, cheap, and reliable data extraction to develop large databases for analysis and academic research, for data such as automated perimetry reports or ophthalmic imaging data (1). Indeed, some of the data can be extracted *via* manufacture-provided licensed software (2), but they are often expensive and can be cost prohibitive for many institutions and practices. Alternatively, study data can be manually transcribed by trained researchers, but this is costly and tedious with high risk for human error (3–5), which limits the types and scope of research projects that can be done.

Static automated perimetry exemplifies this issue well. Perimetry data involves large volume of quantitative data for each location tested, often done serially to track longitudinal progression in conditions such as glaucoma or neuro-ophthalmic disease. Such data can be analyzed using a variety of analysis techniques with both global and localized metrics (6, 7). One challenge in managing the large volume of perimetry data is obtaining accurate and detailed data points from each test (8). Therefore, most recent studies rely on small and single institution datasets containing hundreds of eyes (9). Few studies examining automated perimetry have datasets up to 2,000-3,000 eyes or more, with one study requiring the development of an in-house data extraction software system (10, 11). These studies indicate that there is an unmet need to develop methods to automatically and accurately extract large volume of perimetry studies, which is critical to building massive perimetry datasets for future detection and progression study in the ophthalmology field.

To solve this need in the field of automated perimetry, we have developed and validated a software platform for extraction of Humphrey® Visual Field (HVF) reports, a form of static automated perimetry used widely in clinical environments. Our aim in developing this platform was to automate HVF report data extraction in a fast, accurate way to facilitate (1) development of large-volume datasets for clinical research and (2) novel methodologies in computational analysis of perimetry data.

## Methods

### Description and Development of Platform

The software platform was developed by the author (MS) using Python 3.6.4 (12). The software leverages OpenCV 3.4.3 (13), an open source computer vision library, for image processing and figure detection, Tesseract 4.1.1 (14), an open source optical character recognition library, for metadata extraction, and Fuzzywuzzy (15), a fuzzy regular expression library for text matching. DICOM file reading was done using PyDICOM, an open-source DICOM reading package (16). Development and testing was performed on a MacBook Air (mid-2013) running Catalina 10.15.2 (Apple Inc, Cupertino, CA, USA).

**Figure 1:**
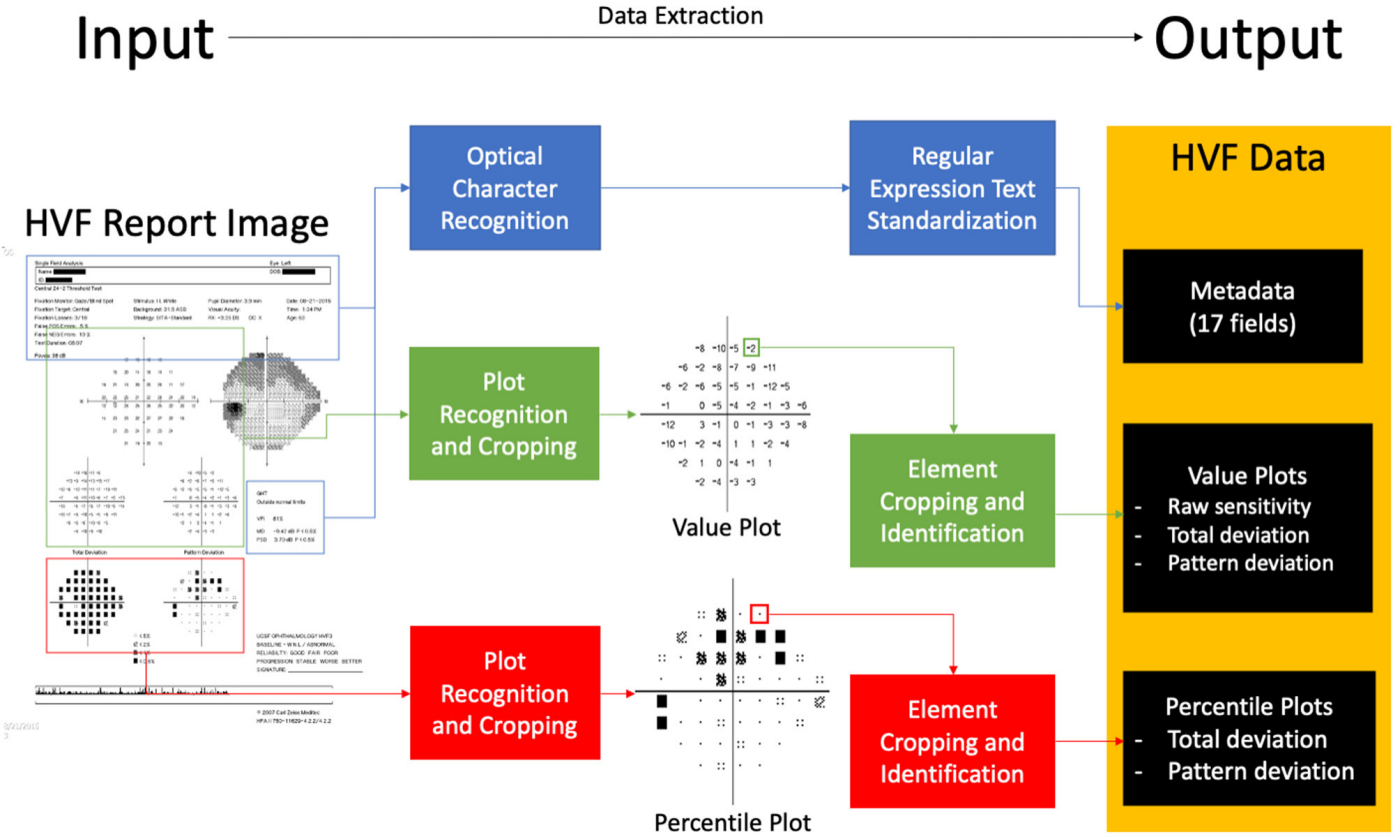
Block diagram of extraction software. An input visual field report identifies areas of metadata, value plots and percentile plots, processes and extracts data, and outputs structured data.

In broad detail, this software platform takes as input HVF report image files, “extracts” data from the report image, and outputs structured, digital data represented in that report (Figure 1). The data on the HVF report image is categorized into three data types: metadata, value plot data, and percentile plot data.

Metadata is defined as any data to be extracted not included within visual field plots. Within HVF reports, 17 fields are identified to be extracted by the platform:

1. Name
2. ID
3. Date of birth
4. Test date
5. Laterality (right or left)
6. Foveal sensitivity
7. False positive rate
8. False negative rate
9. Fixation loss rate
10. Test duration
11. Field size
12. Test strategy
13. Pupil diameter
14. Refraction used
15. Mean deviation
16. Pattern standard deviation
17. Visual Field Index (VFI).

To extract the data, the software first crops the image containing the metadata of interest and applies optical character recognition (OCR) using Tesseract. The resulting text data is then processed using regular expressions and string matching to structure and standardize the text data into the expected metadata fields.

Value plots are defined as plots with numerical perimetry data, that is, raw sensitivity plot data, total deviation value plot data, and pattern deviation value plot data (Figure 1). To extract data, the software locates the plot by identifying the plot axes and subsequently crops the plot image. It then aligns the plot to a 10 × 10 grid, and each cell is processed using a custom-built optical character recognition system (based on template matching) in order to determine and extract the value of the cell.

Percentile plots were defined as plots percentile sensitivity data values, that is, total deviation percentile plot data and pattern deviation percentile plot data (Figure 1). Percentile plots are processed in an identical fashion to value plots, but each cell is processed using a separate template-matching based system to determine the icon of the cell.

Data processed by the platform is represented and stored in an object-oriented format and can be used for further processing within the Python environment.

**Figure 2:**
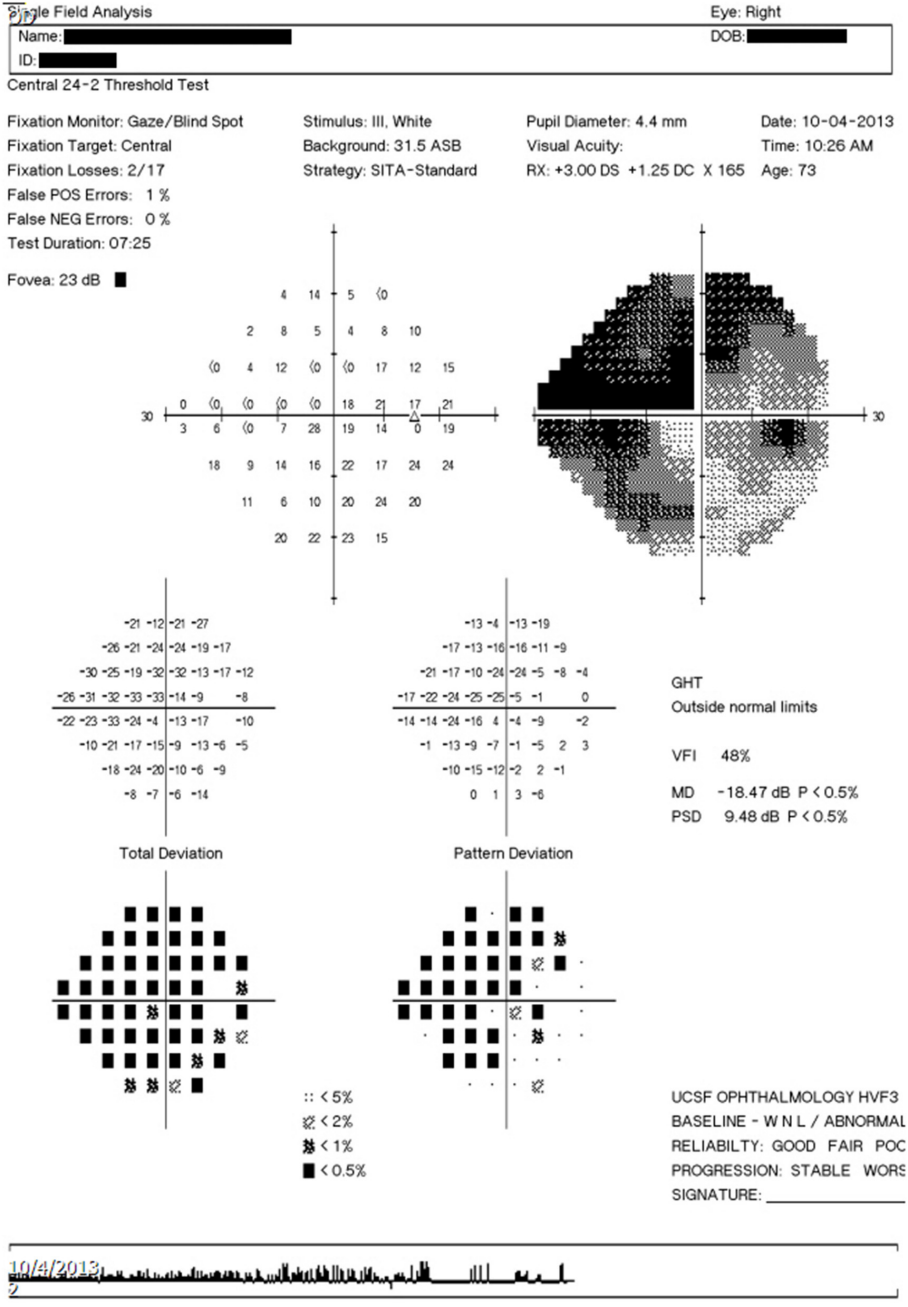
Example output text file. Example output text file corresponding to the image report seen in Figure 3C.

In addition to HVF report images, the software platform can also accept other types of input such as ophthalmic visual field (OPV) DICOM files containing HVF data and text serialization files in Javascript Object Notation (JSON) format that have been outputted by the software platform. An example of the output text file is shown in Figure 2.

The perimetry data processed by the platform can be analyzed and processed internally within the Python environment, output as a JSON text file (e.g., to be re-imported and processed by the software platform at a different time) or output as a tab-delimited file to be imported into a spreadsheet processing software.

The software scripting platform was open-sourced under the GPL 3.0 license (17).

### Extraction Platform Validation

This study was compliant with the Health Insurance Portability and Accountability Act and the Declaration of Helsinki for research involving human participants. Institutional Review Board approval was obtained from the University of California, San Francisco Human Research Protection Program.

#### Visual Field Testing

All VF examinations and reports were done by a Humphrey VF analyzer (HFA2 or HFA3) (Carl Zeiss Ophthalmic Systems, Inc., Dublin, CA) on a 10-2, 24-2 or 30-2 test pattern, size III white stimulus, with a Swedish Interactive Threshold Algorithm (SITA) strategy. Reports were exported as a.PNG image to the ophthalmology department picture archiving and communication system (PACS) server and downloaded from the server.

#### HVF Report Dataset Collection—Selection, Inclusion, and Exclusion Criteria

**Figure 3:**
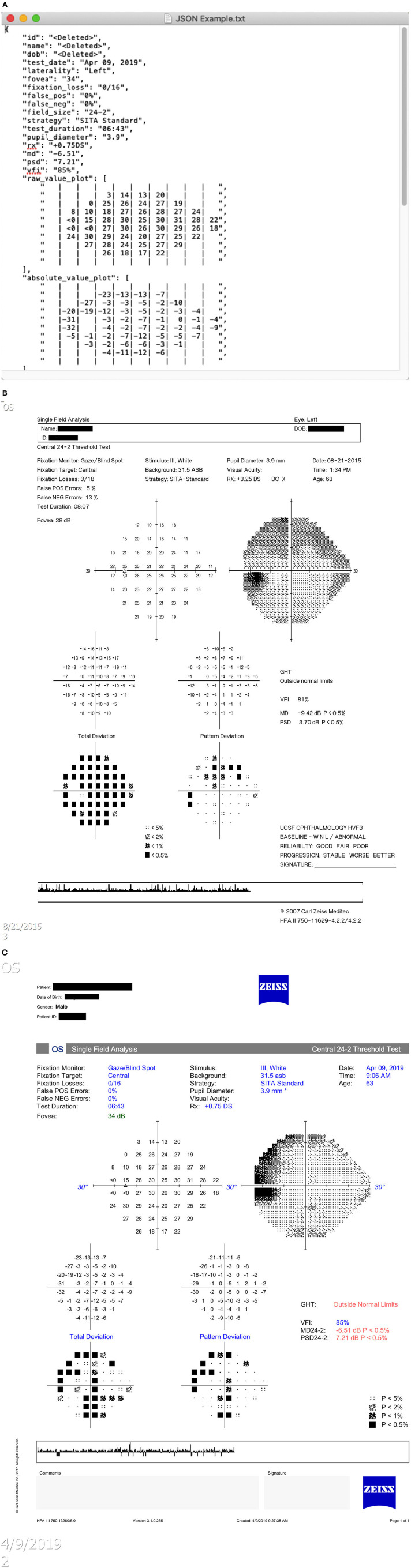
Humphrey Visual Field report layout types. **(A)** Version 1 layout. **(B)** Version 2 layout. **(C)** Version 3 layout.

Three different types of HVF report resolution/layout formats (version 1, 2, and 3 layouts) present in the PACS system of our institution were identified. Examples of these layouts are shown in Figure 3. Image dimensions for these layouts are:

Version 1: 650 pixels by 938 pixels (HFA2, low resolution)
Version 2: 2,400 pixels by 3,180 pixels (HFA2, high resolution)
Version 3: 3,726 pixels by 5,262 pixels (HFA3, high resolution)

A total of 90 HVF report images, with 30 HVFs for each layout version, was collected for validation. The sample size was determined by preliminary extraction tests to ensure valid statistical comparisons. Based on preliminary extraction runs, a human extraction accuracy of 98% and a computer extraction accuracy of 99.3% was assumed. At an alpha level of 0.05 and a power of 90%, assuming a 1:1 study ratio, sample size calculations determined a minimum of 1,808 data points was needed to detect a statistically significant difference; this equates to a minimum of 18 visual field reports. A set size of 30 was chosen to meet and exceed this minimum requirement.

All HVF reports were collected from patients seen at the University of California, San Francisco Ophthalmology Visual Field Testing Clinic. For version 1 layout, 30 historical HVF reports were taken from consecutive patients 2014 or prior. For version 2 layout, 30 HVF reports were selected from consecutive patients seen from March 4, 2019 to March 5, 2019. For version 3, 30 HVF reports taken from consecutive patients seen from August 30, 2019 onward.

A maximum of two HVFs per patient were selected (one for each eye). Only HVFs with strategy SITA-Standard, SITA-Fast or SITA-Faster were included; HVFs performed with a Full-Threshold strategy or any other strategy were excluded. There was no inclusion or exclusion criteria based on patient diagnosis, reliability indices, mean deviation, or type of defect noted.

#### Data Extraction and Accuracy Measurements

Four human extractors, all ophthalmologists familiar with reading HVF reports, were selected. Each extractor manually recorded the data from each HVF report into a spreadsheet, as well as time required for extraction. Each extractor was allowed to perform extraction independently, without proctoring, in an environment they selected as optimal. In addition to manual human extraction, each HVF report image was processed using the data extraction software script.

Each set of extracted data (from human extractors and software extractions script) was compared against data obtained from the DICOM OPV file representing the report of interest, obtained from the Humphrey Field Analyzer device. A custom testing platform, written in Python, was developed to compare these outputs.

Metadata fields were compared on a per-field basis; field were considered correct if the computer image extraction matched exactly to the DICOM reference. Two types of inaccuracy were determined by a masked grader who was blind to human or software data extraction (YH). Formatting inconsistencies were defined as when the extracted data was different from the DICOM reference in a minor way, such that the data still provided correct information; examples include case inconsistencies, whitespace differences, and differences in date reporting. True errors were defined as all other field inequalities that did not represent the correct data.

Data points from value plots and percentile plots were compared on a per data point basis, among all non-empty value data points within value plots. Data points were considered correct if the value from the extraction exactly matched the DICOM reference value.

#### Statistical Analysis

For each HVF record, we calculated the total number of errors for extracting metadata, value plot data, percentile plot by using computer script, and four human extractors. We summarized the errors using total number of errors from all records of each HVF layout (e.g., aggregate errors), aggregate error rate (calculated as aggregate errors divided by the total number of fields tested) and its 95% binomial confidence intervals, and median (inter-quartile) of number of errors in each HVF record. For each of HVF layout, we compared between computer script and each of four human extractors in the mean time used for data extraction using repeated measures one-way analysis of variance and in the number of errors per HVF record using Friedman's Chi-Square test due to skewed distribution. All the statistical analyses were performed in SAS v9.4 (SAS Institute Inc., Cary, NC), and two-sided *p* < 0.05 was considered to be statistically significant.

## Results

The HVF extraction program was developed in line with the specifications outlined in the Methods section. It is available free for access and usage at https://pypi.org/project/hvf-extraction-script/. Its source code can be found at https://github.com/msaifee786/hvf_extraction_script.

Characteristics of the HVF reports for each layout version is shown in Table 1. A total of 1,530 metadata fields, 15,536 value plot data points, and 10,210 percentile data points were tested over three layout version groups. Each group included a similar number of right and left eyes and included at least one report from each field size test. There was representation from each severity of visual field defect based on mean deviation magnitude.

**Table 1:** Characteristics of validation set visual field reports.

	**V1 Layout (*n* = 30)**	**V2 Layout (*n* = 30)**	**V3 Layout (*n* = 30)**
Number of patients	16	16	17
Number of eyes			
Right	15	14	13
Left	15	16	17
Field Size			
24-2	21	24	22
30-2	8	4	6
10-2	1	2	2
Average mean deviation (dB)	−4.81	−3.45	−2.44
>-6.0	24	26	28
−6.0 to −12.0	3	2	1
< −12.0	3	2	1
Average pattern standard deviation (dB)	4.50	2.74	3.43
Total number of metadata fields tested	510	510	510
Total number of value plot data points tested	5,263	5,045	5,228
Total number of percentile plot data points tested	3,453	3,309	3,448

Validation was performed between the computer extraction and human extraction for each HVF layout, measuring extraction times (Table 2), metadata error rates (Table 3A) and format inconsistencies (Table 3B), value plot error rates (Table 4) and percentile plot error rates (Table 5). Notably, minor post-processing editing was done on the human extraction datasets in order to standardized formatting prior to validation testing. Human extractor P2 mislabeled three files in the V1 layout data due to a skip in the sequential numbering; this was corrected prior to the validation comparison. Human extractor P4 skipped a column field in the extracted dataset, which was added in (with blank values) to standardized format prior to validation comparison. Lastly, datasets for P3 and P4 required trivial substitutions of characters (e.g., upper to lower case conversion).

**Table 2:** Extraction times for each resolution layout.

**Extraction Time per report (secs)**	**Computer program (*N* = 30)**	**Human 1 (*N* = 30)**	**Human 2 (*N* = 30)**	**Human 3 (*N* = 30)**	**Human 4 (*N* = 30)**
**V1 layout**					
Mean (SD)	6.0 (0.7)	598.0 (187.4)	1,190.0 (274.8)	886.0 (281.7)	966.0 (228.0)
*P*-value	Reference	<0.001	<0.001	<0.001	<0.001
**V2 layout**					
Mean (SD)	4.9 (0.6)	440.0 (53.0)	846.0 (137.7)	768.0 (201.4)	748.0 (173.1)
*P*-value	Reference	<0.001	<0.001	<0.001	<0.001
**V3 layout**					
Mean (SD)	8.9 (0.8)	394.0 (62.4)	808.0 (150.1)	728.3 (196.2)	708.0 (192.6)
*P*-value	Reference	<0.001	<0.001	<0.001	<0.001

*Extraction times from the computer program was used as reference for all statistical comparisons within each layout*.

**Table 3A:** Comparison between computer program and human metadata extraction (Metadata errors).

**Metadata errors**	**Computer program (*N* = 30)**	**Human 1 (*N* = 30)**	**Human 2 (*N* = 30)**	**Human 3 (*N* = 30)**	**Human 4 (*N* = 30)**
**V1 layout**					
Total errors	18	10	16	24	9
Percentage of total error %^*^	3.5 (2.1-5.5)	2.0 (0.9-3.6)	3.1 (1.8-5.0)	4.7 (3.0-6.9)	1.8 (0.8-3.3)
Median (Q1, Q3) error per report^**^	0 (0, 1)	0 (0, 1)	0 (0, 1)	1 (0, 1)	0 (0, 0)
*P*-value	Reference	0.80	0.56	0.09	0.41
**V2 layout**					
Total errors	6	6	32	47	4
Percentage of total error %^*^	1.2 (0.4-2.5)	1.2 (0.4-2.5)	6.3 (4.3-8.7)	9.2 (6.9-12.1)	0.8 (0.2-2.0)
Median (Q1, Q3) error per report^**^	0 (0, 0)	0 (0, 0)	1 (0, 2)	1 (1, 2)	0 (0, 0)
*P*-value	Reference	0.01	0.001	<0.001	0.046
**V3 layout**					
Total errors	8	7	10	33	1
Percentage of total error %^*^	1.6 (0.7-3.1)	1.4 (0.6-2.8)	2.0 (0.9-3.6)	6.5 (4.5-9.0)	0.2 (0.0-1.1)
Median (Q1, Q3) error per report ^**^	0 (0, 0)	0 (0, 0)	0 (0, 1)	1 (1, 2)	0 (0, 0)
*P*-value	Reference	1.00	0.62	<0.001	0.03

* *Numbers in parenthesis indicate 95% confidence interval*.

** *Q1, Q3 refer to first and third quartile, respectively*.

**Table 3B:** Comparison between computer program and human metadata extraction (format inconsistencies).

**Metadata format inconsistencies**	**Computer Program (*N* = 30)**	**Human 1 (*N* = 30)**	**Human 2 (*N* = 30)**	**Human 3 (*N* = 30)**	**Human 4 (*N* = 30)**
**V1 layout**					
Total number	8	7	7	8	10
Percentage of total inconsistency %^*^	1.6 (0.7-3.1)	1.3 (0.6-2.8)	1.3 (0.6-2.8)	1.6 (0.7-3.1)	2.0 (0.9-3.6)
Median (Q1, Q3) inconsistencies per report^**^	0 (0, 1)	0 (0, 0)	0 (0, 0)	0 (0, 1)	0 (0, 1)
*P*-value	Reference	0.56	0.56	1.00	0.41
**V2 layout**					
Total number	4	2	6	5	9
Percentage of total inconsistency %^*^	0.8 (0.2-2.0)	0.4 (0.1-1.4)	1.2 (0.4-2.5)	1.0 (0.3-2.3)	1.8 (0.8-3.3)
Median (Q1, Q3) number of inconsistencies per report^**^	0 (0, 0)	0 (0, 0)	0 (0, 0)	0 (0, 0)	0 (0, 1)
*P*-value	Reference	0.16	0.41	0.32	0.03
**V3 layout**					
Total number	3	0	5	4	7
Percentage of total inconsistency %^*^	0.6 (0.1-1.7)	0 (0.0-0.7)	1.0 (0.3-2.3)	0.8 (0.2-2.0)	1.4 (0.6-2.8)
Median (Q1, Q3) number of inconsistencies per report^**^	0 (0, 0)	0 (0, 0)	0 (0, 0)	0 (0, 0)	0 (0, 0)
*P*-value	Reference	0.08	0.32	0.56	0.16

* *Numbers in parenthesis indicate 95% confidence interval*.

** *Q1, Q3 refer to first and third quartile, respectively*.

**Table 4:** Comparison between computer program and human on value plot extraction errors.

**Value plot errors**	**Computer program (*N* = 30)**	**Human 1 (*N* = 30)**	**Human 2 (*N* = 30)**	**Human 3 (*N* = 30)**	**Human 4 (*N* = 30)**
**V1 layout**					
Total errors	46	197	603	47	563
Percentage of total error %^*^	0.9 (0.6-1.2)	3.7 (3.3-4.3)	11.5 (10.6-12.4)	0.9 (0.7-1.2)	10.7 (9.9-11.6)
Median (Q1, Q3) errors per report^**^	1.5 (1, 2)	3.5 (2, 8)	2.5 (0, 53)	1 (0, 2)	1.5 (0, 52)
*P*-value	Reference	<0.001	0.16	0.30	0.69
**V2 layout**					
Total errors	2	155	730	48	760
Percentage of total error %^*^	0.0 (0-0.1)	3.1 (2.6-3.6)	14.5 (13.5-15.5)	1.0 (0.7-1.3)	15.1 (14.1-16.1)
Median (Q1, Q3) errors per report^**^	0 (0, 0)	1 (0, 3)	4.5 (0, 54)	1 (0, 2)	2.5 (0, 54)
*P*-value	Reference	<0.001	<0.001	<0.001	<0.001
**V3 layout**					
Total errors	8	118	768	97	653
Percentage of total error %^*^	0.2 (0.1-0.3)	2.3 (1.9-2.7)	14.7 (13.7-15.7)	1.9 (1.5-2.3)	12.5 (11.6-13.4)
Median (Q1, Q3) errors per report^**^	0 (0, 1)	2 (1, 5)	3 (1, 53)	0.5 (0, 3)	2 (0, 52)
*P*-value	Reference	<0.001	<0.001	0.04	<0.001

* *Numbers in parenthesis indicate 95% confidence interval*.

** *Q1, Q3 refer to first and third quartile, respectively*.

**Table 5:** Comparison between computer program and human on percentile plot extraction errors.

**Percentile plot errors**	**Computer program (*N* = 30)**	**Human 1 (*N* = 30)**	**Human 2 (*N* = 30)**	**Human 3 (*N* = 30)**	**Human 4 (*N* = 30)**
**V1 layout**					
Total errors	0	54	302	9	289
Percentage of total error %^*^	0 (0-0.1)	1.6 (1.2-2.0)	8.8 (7.8-9.7)	0.2 (0.1-0.5)	8.4 (7.5-9.3)
Median (Q1, Q3) errors per report^**^	0 (0, 0)	0 (0, 3)	5 (0, 21)	0 (0, 0)	1.5 (0, 24)
*P*-value	Reference	0.003	<0.001	0.08	<0.001
**V2 layout**					
Total errors	0	26	356	38	372
Percentage of total error %^*^	0 (0-0.1)	0.8 (0.5-1.2)	10.8 (9.7-11.9)	1.2 (0.8-1.6)	11.2 (10.2-12.4)
Median (Q1, Q3) errors per report^**^	0 (0, 0)	0 (0, 0)	4 (0, 22)	0 (0, 0)	9 (0, 22)
*P*-value	Reference	0.008	<0.001	0.01	<0.001
**V3 layout**					
Total errors	2	2	435	55	273
Percentage of total error %^*^	0.1 (0.0-0.2)	0.1 (0.0-0.2)	12.6 (11.5-13.8)	1.6 (1.2-2.1)	7.9 (7.0-8.9)
Median (Q1, Q3) errors per report^**^	0 (0, 0)	0 (0, 0)	10 (0, 23)	0 (0, 1)	1 (0, 20)
*P*-value	Reference	1.00	<0.001	0.02	0.002

* *Numbers in parenthesis indicate 95% confidence interval*.

** *Q1, Q3 refer to first and third quartile, respectively*.

### Extraction Times

Average extraction time for the computer platform varied from 4.9 to 8.9 s, with minimal variation between the different layouts (Table 2). The highest resolution V3 layout had the longest average computer extraction time. Human extractors had average extraction times varying from 394 to 1,190 s for all three versions, with a statistically significant longer time in comparison to computer extraction (*p* < 0.001). There was no clear difference in human extraction time among different versions. In general, the computer platform performed extractions on the order of 50-100 times faster than human extractions.

### Metadata Extraction

Within the computer extraction group, there were a total of 32 metadata extraction errors across all three layouts, with a per-layout error rate varying from 1.2-3.5%, with the highest error rate occurring the V1 layout group (Table 3A). The highest frequency of extraction errors was due to incorrect character recognition (seven errors). Among all four human extractors, the average per-layout error rate varied from 2.5-4.4%. Examples of metadata extraction errors that occurred in this study are shown in Table 6.

**Table 6:** Examples of extraction errors.

**Field**	**Extracted Value**	**True Value**	**Type of Error**
**Computer Extractions**			
Metadata			
Test Duration	06:54	06:543	Erroneous extra value
Mean Deviation	0.47	−0.47	Dropped minus sign
Refraction Used	−0.75DS +1.26DC X 88	−0.75DS +1.25DC X 88	Incorrect character recognition
Pupil Diameter	4.1	4.7	Incorrect character recognition
**Value Plot**			
	24	28	Incorrect character recognition
	21	27	Incorrect character recognition
**Human Extractions**			
Metadata			
Mean Deviation	−0.3	−0.39	Missed digit
Test Duration	06:15	06:16	Incorrect character recognition
Refraction Used	−1.5DS DC X	−2.00DS +3.00DC X 175	Incorrect field extracted
Strategy	SITA Standard	SITA Fast	Incorrect field extracted
ID	43150443	34150443	Transposed characters
Date of Birth	11-15-1941	11-16-1941	Incorrect character recognition
**Value Plot**			
	<0	0	Incorrect character recognition
	17	21	Incorrect field extracted

Computer extraction overall performed similarly to human extraction for metadata. In V1 layout, there was no difference between the computer and human extractors. Computer had a lower number of metadata errors than P2 and P3 in V2 layout and P3 in V3 layout, while P4 had less metadata errors than computer in V2 and V3 layouts. There was nearly no significant difference between format inconsistencies between the computer and human extractions in any version layout (Table 3B).

### Value Plot Extraction

For every layout, value plot extraction errors were less for computer extraction than every human extractor (Table 4). These comparisons were statistically significant in layouts V2 and V3. The highest number of value plot errors among human extractors were due to P2 and P4; a large number of these errors occurred due to a frame shift error for all left eyes. Examples of value plot errors that occurred in this study are shown in Table 6.

Computer extraction value plot errors occurred predominantly within the V1 layout extraction; most of the errors occurred as a misidentification between 4, 6, and 8, as well as between 1 and 7 (Table 6). Majority of these occurred in the raw value plot, while the remaining errors occurred in the total deviation value plot. These errors occurred in scattered parts of the plot with no association to a specific location. In the V2 and V3 layout value plot extraction, all errors occurred in the raw value plot along the horizontal midline in the temporal field (i.e., corresponding to the area of the physiologic blind spot). Almost uniformly for these errors, the areas had a reduced sensitivity value (often “0” or “ <0”) and an adjacent open triangle icon (or fragment thereof) near the value.

### Percentile Plot Extraction

Overall, percentile plot extraction errors occurred rarely in the computer extraction (Table 5). No computer extraction percentile plot extraction errors occurred in the V1 and V2 layout. Two errors occurred in the V3 layout in total deviation percentile plots. The computer performed nominally lower than every human extractor for every layout; all but two of these comparisons (P3 in V1 and P1 in V3) were statistically significant.

## Discussion

To our knowledge, this is the first open-source data extraction software script for perimetry output in the literature. The main purpose of the development of this platform is to improve our ability to research and analyze perimetry data and ultimately to better guide treatment of vision-threatening diseases. To that end, this code has been made available through the Python Package Index (PyPi), and its source code has been published as open source, available through GitHub. We encourage anyone to utilize this program, scrutinize its effectiveness, improve upon it and adapt it for their own uses.

The method employed to extract data from HVF perimetry reports in this script is optical character recognition (OCR) technology, which has been available since the 1950s (18). Recently, this technology has improved significantly with improved image processing techniques and the advent of neural networks. In the literature, studies that have specifically used OCR technology for medical data exaction tasks mostly focus on scanned reports for clinical laboratory tests, with reasonably high accuracy (19–21). Adamo et al. utilized Tesseract OCR (the same OCR platform as used in our script) to achieve an accuracy of 95% in their extraction system (19). Another team was able to achieve a similar accuracy of 92.3-95.8% using a custom neural network model on multilingual reports containing Chinese and Latin characters (20, 21). Our script shows a nominally higher accuracy rate than these systems; this is likely due to our study utilizing standardized digital report images rather than scanned documents. Nonetheless, these studies highlight the value of computer vision and OCR tools in the data extraction of medical reports.

Our script offers specific value in ophthalmology, especially in the field of glaucoma, by facilitating access to structured perimetry data. Static automated perimetry is an integral component in the management and monitoring of glaucoma, and numerous studies in the literature have examined various perimetry metrics in search of an optimal marker of diagnosis or progression (22). In recent years, machine learning and neural networks have also been used in perimetry research (9); these algorithms are heavily dependent on well-categorized, large volume datasets. Thus, developing new perimetry metrics is an important focus of research in glaucoma (23), and access to structured perimetry is critical in facilitating this research (23). Our program was designed to offer a versatile option to generate structured HVF data for analysis from DICOM files or images files (such as JPG or PNG formats). With this, the program can serve as an avenue to several opportunities for perimetry data analysis. Additionally, this platform can potentially be used in conjunction with other analysis platforms such as the R package visualField (an open source module for analysis of visual field data), with the appropriate software to interface the two systems (24). Our platform has been used in a published study on HVFs in glaucoma patients undergoing glaucoma tube shunt implantation (25). Other research teams have performed studies with large volumes of HVFs for metric analysis and machine learning using in-house extraction software (6, 11); however, their script was not published and validation cannot be compared with ours.

One of the main strengths of computer extraction is the speed of extraction. Not only does the computer script offer more than a 50-fold increase in extraction speed, but also allows the extraction process to be automated for a large number of reports. Thus, the computer script can free up researchers for other tasks, and overall help reduce the cost and effort of data extraction. In institutions where structured digital perimetry data are not easily available straight from the acquisition devices, the computer extraction script offers an effective alternative to costly human extraction.

The validation results show an overall low error rate for the computer extraction data. Most errors occurred in metadata extraction, which has the most variability in the type and structure of the extracted data fields. As expected, the error rate increases with lower resolution images; this is due to the nature of image detection and OCR technology, which we used heavily in metadata extraction. Despite this correlation, metadata error rates remain low and similar to human extraction error rates, regardless of resolution of input image.

The error rate for computer extracted value and percentile data was very low and were statistically significantly better than human extraction except for value plot extractions in the low-resolution layout V1. Misidentification of similar appearing numbers in low-resolution images and interference of the open triangle icon in the area of the physiologic blind spot within the raw sensitivity plot were the main reasons for errors. The accuracy of the computer script in value and percentile plot data shows one of its main strengths, especially in the face of significant error rates in human extraction.

A notable result in our validation study is the high frequency of errors that arises from manual, human data extraction. Data errors in medical research have been studied in the past; one study showed error rates ranging from 2.3-26.9% in separately maintained clinical research databases at a single institution, due to a combination of presumed transcription and cognitive errors (26). This compares similarly to our study, with human extraction error rates as high as 10-15% in some categories. The substantially high error rate among human extraction in our study is possibly related to the display of plot data within HVF reports, which contain a high density of values within an area. This is supported by prior studies that show that displaying a high volume of data in the source document is correlated with transcription errors (4). Additionally, human extraction data tends to be variably formatted, especially when several different people contribute to the extracted datasets; this variability of data often requires standardization prior to further processing. Overall, understanding the relative strengths and weaknesses of human vs. computer extraction is important to improving research data integrity.

Lastly, it should be noted that while the computer program extraction is faster and more accurate than human extraction, it does not have 100% accuracy. Human validation of the extracted data may be needed to correct any computer errors. Understanding the limitations of computer data extraction and common areas of errors can help guide human validation of the data to speed up the process.

There are a few limitations of this validation study. First, the report layouts were limited to three distinct resolutions; while the different resolutions demonstrate the correlation of accuracy with resolution, the limited resolution layouts may not capture the full spectrum of image resolutions in use in the community. The limited number of reports per trial and selection methodology may not fully represent the spectrum of visual field defects possible, which may limit the generalizability of the error rates to specific HVF reports.

In summary, in this paper we introduce and validate a computer program for the extraction of HVF data from report images. In comparison to human extraction, computer extraction is faster and more accurate; however, human validation of the computer extraction data may be necessary for situations that require high fidelity of data. Overall, this program can help reduce the cost of data analysis for research institutions where HVF data is otherwise inaccessible.

## Data Availability Statement

The original contributions presented in the study are included in the article/supplementary material, further inquiries can be directed to the corresponding author/s.

## Ethics Statement

This study was compliant with the Health Insurance Portability and Accountability Act and the Declaration of Helsinki for research involving human participants. Institutional Review Board approval was obtained from the University of California, San Francisco Human Research Protection Program.

## Author Contributions

MS developed software platform and contributed to study design, data collection, and analysis. JW, YL, PM, and JP contributed to data collection and analysis. YY and G-SY contributed to data analysis and statistical calculations. YH contributed to study design, data analysis, and statistical calculations. All authors contributed to manuscript preparation.

## Conflict of Interest

The authors declare that the research was conducted in the absence of any commercial or financial relationships that could be construed as a potential conflict of interest.
